# Exploring the therapeutic potential of metformin on cognitive function, amyloid pathology, and microglial homeostasis in Alzheimer's disease models

**DOI:** 10.1002/alz70855_102986

**Published:** 2025-12-23

**Authors:** Nicolas Gabriel Gonzalez Perez, Carlos Javier Pomilio, Luciano Arcucci, Natali Rasetto, Melisa Bentivegna, Melina Paula Bellotto, Jessica L Presa, Soledad Porte Alcon, Juan Beauquis, Flavia Saravia

**Affiliations:** ^1^ CONICET, Buenos Aires, Argentina; ^2^ University of Buenos Aires, Buenos Aires, Argentina; ^3^ CONICET, Buenos Aires, Buenos Aires, Argentina

## Abstract

**Background:**

Alzheimer's Disease (AD) and type 2 diabetes mellitus (T2D) share an exacerbated neuroinflammatory response coordinated by microglial cells and a decreased autophagic flux. Treatment with the normoglycemic metformin (MET) has been associated with reduced microglial activation and restored autophagy in T2D models. Our objective, within the framework of drug repositioning, is to evaluate the therapeutic potential of MET on experimental AD using mouse models and in vitro experiments.

**Method:**

We treated 9‐month‐old male PDAPP‐J20 mice carrying AD mutations with MET (350 mg/kg i.p. three times a week for three weeks) or vehicle and we assessed cognitive profile using spatial memory tests such as the Barnes maze. From brain sections containing hippocampus, we analyzed the impact of MET on amyloid pathology, neurogenesis status by evaluation of the new‐born neuron marker DCX and microglial status. In vitro, the murine BV2 microglial cell line was exposed to 0.05 µM of Aβ 1‐42 for 24 hours, followed by 1 hour of treatment with 0.2 mM MET, and autophagic flux was evaluated under these conditions.

**Result:**

In MET‐treated mice we found an improvement in spatial memory and hippocampal neurogenesis and a decrease in amyloid pathology compared to vehicle‐treated group. AD mice exhibited a decrease in the homeostatic microglial marker TMEM119, which was reversed by MET treatment. Additionally, a restoration of microglial autophagic flux was observed, evidenced by a reduction in the autophagosome marker p62 in transgenic mice treated with MET. In vitro, exposure to amyloid peptides resulted in a blockade of autophagic flux, assessed by p62 accumulation using Bafilomycin, which was reversed by MET.

**Conclusion:**

Our results indicate that metformin may have a therapeutic effect in AD models restoring cognitive performance. This effect appears to be due to a microglial gain of function by rehabilitating the autophagic flux blocked by amyloid peptides. Metformin may also contribute to the reduction of amyloid pathology and improvement of hippocampal neurogenesis.

